# Predictors of Walking App Users With Comparison of Current Users, Previous Users, and Informed Nonusers in a Sample of Dutch Adults: Questionnaire Study

**DOI:** 10.2196/13391

**Published:** 2021-05-12

**Authors:** Gert-Jan De Bruijn, Joan Martine Dallinga, Marije Deutekom

**Affiliations:** 1 Amsterdam School of Communication Research (ASCoR) University of Amsterdam Amsterdam Netherlands; 2 Department of Physiotherapy Faculty of Health Amsterdam University of Applied Sciences Amsterdam Netherlands

**Keywords:** technology, walking, health, adult, survey, questionnaires

## Abstract

**Background:**

The last decade has seen a substantial increase in the use of mobile health apps and research into the effects of those apps on health and health behaviors. In parallel, research has aimed at identifying population subgroups that are more likely to use those health apps. Current evidence is limited by two issues. First, research has focused on broad health apps, and little is known about app usage for a specific health behavior. Second, research has focused on comparing current users and current nonusers, without considering subgroups of nonusers.

**Objective:**

We aimed to provide profile distributions of current users, previous users, and informed nonusers, and to identify predictor variables relevant for profile classification.

**Methods:**

Data were available from 1683 people who participated in a Dutch walking event in Amsterdam that was held in September 2017. They provided information on demographics, self-reported walking behavior, and walking app usage, as well as items from User Acceptance of Information Technology, in an online survey. Data were analyzed using discriminant function analysis and multinomial logistic regression analysis.

**Results:**

Most participants were current walking app users (899/1683, 53.4%), while fewer participants were informed nonusers (663/1683, 39.4%) and very few were previous walking app users (121/1683, 7.2%). Current walking app users were more likely to report walking at least 5 days per week and for at least 30 minutes per bout (odds ratio [OR] 1.44, 95% CI 1.11-1.85; *P*=.005) and more likely to be overweight (OR 1.72, 95% CI 1.24-2.37; *P*=.001) or obese (OR 1.49, 95% CI 1.08-2.08; *P*=.005) as compared with informed nonusers. Further, current walking app users perceived their walking apps to be less boring, easy to use and retrieve information, and more helpful to achieve their goals. Effect sizes ranged from 0.10 (95% CI 0.08-0.30) to 1.58 (95% CI 1.47-1.70).

**Conclusions:**

The distributions for walking app usage appeared different from the distributions for more general health app usage. Further, the inclusion of two specific subgroups of nonusers (previous users and informed nonusers) provides important information for health practitioners and app developers to stimulate continued walking app usage, including making information in those apps easy to understand and making it easy to obtain information from the apps, as well as preventing apps from becoming boring and difficult to use for goal attainment.

## Introduction

### Background

The immense popularity of smartphones in the past decade has led to a large number of mobile health apps for those smartphones. Through those apps, people can receive relevant and personalized information and feedback on progress toward public health goals (eg, eating five portions of fruit and vegetables a day, 10,000 steps per day) or self-constructed goals (eg, 10 extra flights of stairs). Mobile health apps tend to focus on physical activity patterns, arguably because the built-in GPS of smartphones allows for an unobtrusive way to monitor those activity patterns. Mirroring the increase in the popularity of mobile health apps, substantial research has investigated the applicability of those health-related mobile apps to influence health behavior in intervention studies or to understand demographic, behavioral, and psychological predictors of mobile health app usage [1-5].

### Intervention Effects of Mobile Health Apps

Intervention studies through mobile apps have focused on a wide range of health-related behaviors, such as the treatment of alcohol use disorders [6], weight reduction [3], and physical activity promotion [3,5,7-9]. For physical activity specifically, summary studies have reported positive effects of mobile apps on physical activity [3-5,7]. For instance, Zhao et al [4] found that 17 out of 23 eligible mobile app intervention studies reported positive effects on physical activity behaviors. These findings echo earlier findings from Bort-Roig et al [5], who found that four out of five physical activity mobile app intervention studies increased physical activity, while Fanning et al [7] performed a meta-analysis of 11 mobile app physical activity intervention studies and found a moderate-to-large effect size increase in physical activity for interventions using mobile apps when compared with control conditions. Thus, mobile app interventions appear to be effective when it comes to increasing physical activity behaviors, although some recent systematic reviews have been somewhat less supportive [9-11].

### Predictors of Mobile Health App Usage

For predictors of mobile health app usage, most studies have commonly reported that mobile health app users are younger, have higher education, are more involved with or conscious of a healthy lifestyle, and have higher levels of health literacy [1,2,12,13]. For instance, in a population-based survey study among German adults, Ernsting et al [2] found that 20% of smartphone users used health apps on their smartphones and that, compared with nonusers, health app users were younger and more health literate. In addition, health app users were engaging in healthier behaviors. In a similar study on health app usage in South Korea, Cho et al [12] surveyed 765 young adults and found that higher health app usage and stronger perceived efficacy to use health apps were associated with younger age and higher eHealth literacy. Finally, in a population-based survey in over 3500 US adults, Carroll et al [1] found that health app users were younger and had higher education and income. Further, participants who used health apps were more likely to meet physical activity recommendations and have a more positive intention to be physically active. Most study results to date have been mixed regarding the effects of gender on health app usage, with some studies finding gender effects [1] and some not finding these effects [12,13]. Thus, mobile health app users are likely to be younger, have higher education, and be more involved with health-related actions than nonusers.

### Current Caveats in Mobile Health App Studies

Despite the informative nature of the current evidence base, there are two important caveats that require further research attention to better understand mobile health app usage. First, most studies on the predictors of mobile health app usage have focused on a simple dichotomy of users versus nonusers. For instance, Carroll et al [1] categorized participants from the 2015 Health Information National Trends Survey in the United States not only along the lines of having a mobile device (ie, mobile or tablet), but also according to whether participants had a health app on their devices. Likewise, Ernsting et al [2] used data from a German population-based sample and compared not only smartphone users and nonusers, but also health app users and nonhealth app users among smartphone users. Such dichotomizations seem to ignore the waxing and waning of a wide range of human health-related actions [14-16], including physical activity [17]. For instance, Conroy et al [17] employed an ecological momentary assessment design among young adults across a 10-week period and found substantial within-person variations in both physical activity intentions and behaviors. These variations presumably also occur for media use [18], but there is remarkably little investigation into specific subgroups of nonusers of mobile health apps. To our knowledge, only one study further divided nonusers into disengaged nonusers (ie, people who have previously used health apps, but no longer use them), informed nonusers (ie, people who have thought about using health apps, but decided not to use them), and unengaged nonusers (ie, people who have never thought about using health apps) [19]. Among the 765 nonusers of apps in this previous study [19], the largest proportions of nonusers were unengaged nonusers (n=301, 39.3%) and informed nonusers (n=214, 28.0%), while a smaller proportion was disengaged users (n=155, 20.2%), with the remaining 95 (12.4%) participants being categorized as participants who decided to start using apps, but were currently not yet using them. Importantly, there were considerable differences between these nonusers in age, with unengaged nonusers being older than most other nonusers. Further, unengaged nonusers reported a healthier eating style than current users, but no differences in eating style emerged between current users and other nonuser profiles. Thus, further differentiation into more specific groups of nonusers seems to be a fruitful research approach to understand the characteristics of subgroups that decide to stop or start using health apps.

A second caveat of the current evidence base is that there is a strong focus on either generic health apps, such as those developed for smoking cessation, dietary change, medication intake, and physical activity [2,12], or apps that focus on a specific behavioral domain, such as general physical activity or dietary intake [20]. However, an investigation of app usage in such a broad behavioral domain tends to neglect various important research findings that have demonstrated poor predictability on broad outcome measures [21] and neglect theoretical suggestions from behavioral prediction models that tend to require a focus on specific goals and behaviors, such as goal-setting theory [22] and the theory of planned behavior [23]. Moreover, research into the determinants of physical activity behaviors has demonstrated not only that people are more motivated to engage in low-to-moderate physical activities than in vigorous physical activities, but also that motivation-physical activity relationships differ across the levels of physical activity intensity [24,25]. Thus, the current focus in mobile health app usage research on broad behavioral domains (ie, physical activity) may obscure important differences that exists for more specific behaviors in these domains (ie, strenuous exercise versus brisk walking).

### Aim

The purpose of this study was threefold. First, we aimed to describe distributions of walking app usage in a sample of Dutch adults, with a specific focus on two important subgroups of nonusers. Because research has indicated that one of the largest subgroups of nonusers is informed nonusers [19], a key focus was to include this subgroup. Likewise, disengaged nonusers represent an important target group, because (in contrast to informed nonusers) they have had previous app experience, but have decided to not continue using the apps. Consequently, they may provide important information on why some people decide to continue using apps and why some do not. Second, we included not only demographic and behavioral correlates as predictors of these subsamples, but also media-use elements of app usage. Media-use elements relate to the experiences that people have when using a specific app [26] or a priori expectations of apps they intend to use [27]. Although media-related experiences [28] are known to be key predictors of continued media usage [29], including health-related media [30] and apps [27], most research on understanding health app usage has focused on either behavioral or demographic correlates, or such factors as health literacy [1,2,19]. Third, we focused on a specific health-related behavior, namely recreational walking. Walking is key to protect against various physical ailments and is associated with various benefits, such as decreased risk factors in type 2 diabetes patients [31] and decreased cardiovascular risk in the general population [32]. Thus, the main aim of this study was to investigate the distributions and predictors of app usage for a specific health behavior, namely walking.

## Methods

### Participants and Procedures

Data for this study were collected from participants of the Dam tot Dam Wandeltocht (Dam-To-Dam Walk), a walking event in September 2017. This recreational event was organized in Amsterdam, the Netherlands. The inclusion criteria were being 18 years or older and having signed the informed consent form. Three days after the Dam tot Dam Wandeltocht, the target population of the survey (all participants of the Wandeltocht) was invited by email to participate in an online survey that was coordinated by SurveyMonkey [33], using the email address participants provided for their Dam-To-Dam Walk registration. No other announcements or advertisements were made to advertise the study. The email contained a link to a closed online survey, which started with a brief introduction of the study. This introduction provided information about the aim of the study, the right of the participants to quit at any time, and the criteria to participate in the study. After reading the introduction, participants who volunteered to participate in the study provided informed consent. The survey took approximately 15 to 20 minutes to complete, and participants received no reward for survey completion. The timeframe of the survey was September 15, 2017, until October 3, 2017. One week after the first invitation, a reminder email was sent to people who had not responded yet. One week after that, the survey was closed. Participants’ IP addresses were registered to prevent people from completing multiple surveys. No other personal information was collected, and IP address data were anonymized. For this study, ethical approval was not obligatory.

### Measures

A nonvalidated survey was used to collect data on study variables, which were partly based on previous studies [13,34,35]. Depending on walking app usage status, the survey was either six (current or previous user) or three (informed nonuser) pages long. The number of questions per survey page ranged from one to three, whereas the number of response items per question ranged from one to eight. Most questions did not have a “rather not say” response option, and participants were able to move between survey pages to modify previous answers. The questions and items were not randomized. Two independent researchers outside the author team tested the survey regarding usability and functionality. Data completeness was checked by the first author.

Educational attainment was assessed by presenting participants with a list of commonly completed educational levels in the Netherlands (eg, elementary school, lower vocational education, and university degree). Participants were also able to write down their educational attainment if this list did not include their attained education. The definition from Statistics Netherlands was used to categorize participants as having either low, middle, or high education [36]. Low education was defined as educational attainment ranging from the completion of primary school to the completion of junior classes in secondary education, middle education was defined as the completion of secondary education and middle vocational training, and high education was defined as the completion of higher vocational training, and bachelor and master programs. BMI was calculated from self-reported height and weight following World Health Organization guidelines [37]. Participants were categorized into normal weight, overweight, or obese groups based on those guidelines.

Self-reported walking behavior was assessed by having participants indicate how often they had engaged in recreational walking (1, less than once per month; 2, one to three times per month; 3, once per week; 4, twice per week; 5, three times per week; 6, four or five times per week; 7, more than five times per week), as well as the average walking duration of each bout (1, 0 to 30 minutes; 2, 30 minutes to 1 hour; 3, 1 hour to 90 minutes; 4, 90 minutes to 2 hours; 5, 2 hours to 2.5 hours; 6, 2.5 hours to 3 hours; 7, longer than 3 hours). Participants who reported engaging in recreational walking at least 5 days per week and longer than at least 30 minutes per bout were categorized as meeting the public health guidelines for moderate physical activity [38].

Walking app usage was assessed by asking participants to tick one of the following four options: (1) I currently use a walking app; (2) I do not currently use a walking app, but I have used one previously; (3) I know of walking apps, but I do not use them; and (4) I do not know of walking apps and I do not use them. Those who used a walking app were further asked to indicate the use duration (1, less than 3 months; 2, 3 to 6 months; 3, 7 to 12 months; 4, 1 to 2 years; 5, 2 to 3 years; 6, 3 to 4 years; 7, 4 to 5 years; 8, longer than 5 years) and frequency (1, all my walking bouts; 2, most of my walking bouts; 3, half of my walking bouts; 4, several of my walking bouts). For frequency, participants were classified as either “always app users” or “fewer than always app users.” For duration, participants were classified into three categories (1, duration of 6 months or less; 2, duration between 6 and 12 months; 3, duration longer than 1 year).

App usage experiences were assessed with five items derived from the “User Acceptance of Information Technology” model [34,35]. Participants indicated their agreement (−2, totally disagree; +2, totally agree) with the following items: (1) I find it easy to understand how the app works; (2) It takes a lot of time to understand the app; (3) I find it easy to get information from the app; (4) I find it boring to use the app; and (5) It is easy to achieve my goal with the app. Previous and current users were asked to consider the walking app they used most often, and informed nonusers were asked to consider walking apps in general.

### Analysis Plan

#### Distributions of Walking App Users and Categorical Predictor Variables of Walking App Usage

To investigate univariate distributions of the categorical predictors (gender, weight status, educational attainment, meeting physical activity recommendations, walking app frequency, and walking app duration) across the three app groups, chi-square tests were performed. To investigate the multivariate effect of these same categorical predictors, multinomial logistic regression analysis was performed to calculate the odds of being (1) a current user (reference category), (2) a previous user, and (3) an informed nonuser from gender, education, weight status, and walking behavior. We also investigated the odds of being a current user versus a nonuser, where previous users and noninformed users were collapsed and grouped. Recommended cutoff points for effect sizes for odds ratios (ORs) were followed [39].

#### Continuous Predictor Variables of Walking App Usage

To predict profile classification from continuous predictors (app experiences, age, and BMI), discriminant function analysis (DFA) was performed. DFA involves a multivariate test to predict group membership based on linear combinations of continuous predictor variables. DFA not only presents the proportion of correctly classified participants, but also reports the correlations of each predictor variable for the discriminant functions. These correlations can be used to interpret the relevance of predictor variables for group membership, with the first function containing the most discriminating predictors. The DFA was followed up by a multivariate analysis of variance, including post-hoc tests using Bonferroni correction and calculation of Cohen *d* [40] to assess the effect sizes of the mean score differences.

## Results

### Participant Characteristics

**Figure 1:**
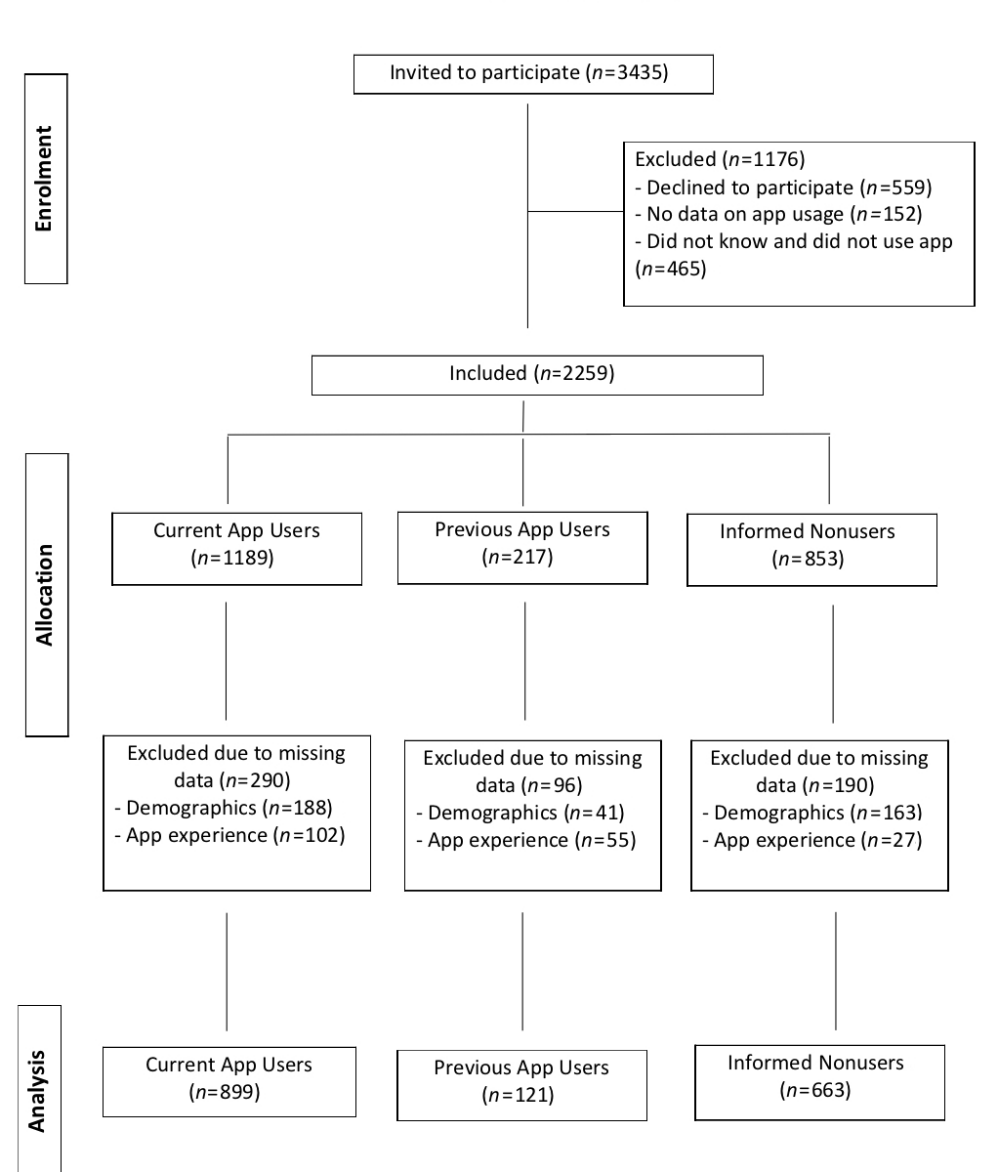
Study flowchart.

Figure 1 presents a flowchart from study invitation to data analysis, with 3435 participants invited to participate. Of those invited, 559 (16.2%) participants declined to participate, 152 (4.4%) did not report any data after accepting the invite, and 465 (13.5%) did not know of mobile walking apps and did not use them. These latter participants were excluded for the purpose of this study. During the remainder of the survey, a further 576 of 2259 (25.5%) participants did not provide information on app use experiences (184/2259, 8.1%) or on demographics or BMI (392/2259, 17.4%). Therefore, the final sample for the main analyses included 1683 participants.

Table 1 presents the characteristics of the total sample and the three profiles. Of the 1683 participants, most were female (1250/1683, 74.3%) and had a middle (758/1683, 45.0%) or high (645/1683, 38.3%) educational level. The mean age was 51.0 years (SD 11.6), and the mean BMI was 26.0 kg/m^2^ (SD 4.5). Most participants had a normal weight (772/1683, 45.9%) or were overweight (679/1683, 40.3%), and a small proportion (232/1683, 13.8%) was obese. A little over a fifth of the sample (358/1683, 21.3%) reported walking at least 3 days per week for a minimum of 30 minutes per session. Over half (899/1683, 53.4%) reported currently using an app for walking. Little under 10% (121/1683, 7.2%) reported having previously used an app for walking, and little over a third (663/1683, 39.4%) reported knowing about walking apps but not using them. Among the current and previous users (1020/1683, 60.6%), 558 (54.7%) reported always using a walking app when walking, and nearly two-thirds of users (662/1020, 64.9%) had been using an app for longer than a year.

**Table 1:** Distributions of categorical variables for the total group and for each of the profiles.

Variable		Total (n=1683), n (%)	Current users (n=899), n (%)		Previous users (n=121), n (%)		Informed nonusers (n=663), n (%)		χ^2^ (*df*)		*P* value		
**Gender**									2.1 (2)		.35		
	Female	1250 (74.3)	652 (72.5)	96 (79.3)		502 (75.7)							
	Male	433 (25.7)	247 (27.5)	25 (20.7)		161 (24.3)							
**Education**									1.4 (4)		.84		
	Low	280 (16.6)	148 (16.5)	22 (18.2)		110 (16.6)							
	Middle	758 (45.0)	398 (44.3)	57 (47.1)		303 (45.7)							
	High	645 (38.3)	353 (39.2)	42 (34.7)		250 (37.7)							
**Weight status**									12.4 (4)		.02		
	Normal	772 (45.9)	395 (43.9)	46 (38.0)		331 (49.9)							
	Overweight	679 (40.3)	363 (40.4)	54 (44.6)		262 (39.5)							
	Obese	232 (13.8)	141 (15.7)	21 (17.4)		70 (10.6)							
**Walking behavior^a^**									6.3 (2)		.04		
	Meets recommendations	358 (21.3)	208 (23.1)	34 (28.1)		116 (17.5)							
	Does not meet recommendations	1325 (78.7)	691 (76.9)	87 (71.9)		547 (82.5)							
**Walking app use frequency^b^**									55.2 (1)		<.001		
	Always	559 (54.6)	530 (59.0)	28 (23.1)		N/A^c^							
	Less than always	464 (45.4)	369 (41.0)	93 (76.9)		N/A							
**Walking app use duration^b^**									40.5 (2)		<.001		
	6 months or less	241 (23.6)	184 (20.5)	55 (45.5)		N/A							
	6-12 months	119 (11.6)	103 (11.5)	16 (13.2)		N/A							
	More than 1 year	663 (64.8)	612 (68.1)	50 (41.3)		N/A							

^a^Defined as walking at least 5 days per week and at least 30 minutes per day.

^b^Assessed for current and previous users only.

^c^N/A: not applicable.

### Categorical Predictor Variables of Walking App Usage

There were significant differences in the distributions of the categorical variables over the three profiles for weight status, walking behavior, and (for current and previous users only) walking app frequency and duration. Specifically, lower proportions of obese participants were found among informed nonusers (70/663, 11%) than among previous (21/121, 17%) or current users (141/899, 15.7%). Likewise, there were lower proportions of participants meeting the activity recommendations among informed nonusers (547/663, 82.5%) than among previous (87/121, 72%) or current users (691/899, 76.9%). Finally, there were higher proportions of people who always used walking apps during their walking routine among current users (530/899, 59.0%) than among previous users (28/121, 23%).

The multinomial logistic regression revealed a significant final model (−2 log likelihood=276.96, χ^2^_12_=30.79, *P*=.002). Meeting the recommendations for physical activity (−2 log likelihood=288.27, χ^2^_2_=11.31 *P*=.004) and weight status (−2 log likelihood=291.84, χ^2^_4_=14.88, *P*=.005) were significant contributors to this model, whereas gender (*P*=.13) and educational attainment (*P*=.872) were not significant contributors. Follow-up tests showed that there were no relevant differences between current users and previous users. As compared to informed nonusers, current users were more likely to report having walked at least 5 days per week and for at least 30 minutes per bout (OR 1.44, 95% CI 1.11-1.85; *P*=.005) and more likely to be overweight (OR 1.72, 95% CI 1.24-2.37; *P*=.001) or obese (OR 1.49, 95% CI 1.08-2.08; *P*=.005). Explained variance was low (Nagelkerke R^2^=0.02).

The binary logistic regression analysis, where previous users and informed nonusers were collapsed and grouped as nonusers and compared with current users, revealed a similar pattern, with current users being more likely to report having walked at least 5 days per week and for at least 30 minutes per bout (OR 1.29, 95% CI 1.02-1.63; *P*=.04). Nonusers were more likely to have a normal weight as compared to users (OR 1.18, 95% CI 1.02-1.36; *P*=.02). Explained variance was low (Nagelkerke R^2^=0.01).

### Continuous Predictor Variables of Walking App Usage

There were two significant discriminant functions (function 1: χ^2^_14_=1033.22, *P*<.001, canonical correlation=0.68, Wilk λ=0.54; function 2: χ^2^_6_=15.03, *P*=.02, canonical correlation=0.09, Wilk λ=0.99). These functions correctly classified 71.5% of cases. Table 2 presents the standardized discriminant coefficients for these functions, the mean scores and standard deviations for each of the predictor variables across the three groups, and the post-hoc comparisons and Cohen *d* for these differences. For the first discriminant function, all app usage coefficients were larger than 0.30, with the strongest predictors being “app boredom” (*r*=0.87), “goal attainment” (*r*=−0.63), “app info retrieval” (*r*=−0.44), and “ease of app usage” (*r*=0.33). For the second discriminant function, age (*r*=0.54) and BMI (*r*=−0.78) were the strongest predictors.

**Table 2:** Discriminant function analysis.

Predictors	F1^a^	F2^b^	1. Current users, mean (SD)^c^	2. Previous users, mean (SD)^c^	3. Nonusers, mean (SD)^c^	*F*_2, 1680_	*P* value	η^2^	Post-hoc
Easy to understand the app	−0.33	0.33	1.0 (0.8)	0.7 (1.0)	0.4 (0.9)	78.3	<.001	0.09	1>2, *P*=.01 1>3, *P*<.001 2>3, *P*<.001
Takes time to understand the app	0.52	−0.19	−1.2 (0.8)	−0.7 (0.9)	−0.4 (0.9)	189.6	<.001	0.18	1>2, *P*<.001 1>3, *P*<.001 2>3, *P*<.001
Easy to retrieve info from the app	−0.44	0.33	0.9 (0.8)	0.6 (0.9)	0.2 (0.8)	135.3	<.001	0.14	1>2, *P*<.001 1>3, *P*<.001 2>3, *P*<.001
Boring to use the app	0.87	0.23	−1.2 (0.8)	−0.4 (0.9)	0.0 (0.7)	530.3	<.001	0.39	1>2, *P*<.001 1>3, *P*<.001 2>3, *P*<.001
Easy to achieve my goal with the app	−0.63	0.04	0.7 (0.8)	0.1 (0.9)	−0.3 (0.7)	275.7	<.001	0.24	1>2, *P*<.001 1>3, *P*<.001 2>3, *P*<.001
BMI	−0.08	0.54	26.2 (4.5)	26.7 (4.9)	25.5 (4.4)	6.4	.002	0.01	1=2, *P*=.95 1>3, *P*=.005 2>3, *P*=.03
Age	0.13	−0.78	49.8 (11.3)	48.5 (11.9)	52.9 (11.7)	16.9	<.001	0.02	1=2, *P*=.71 1>3, *P*<.001 2>3, *P*<.001

^a^F1 reflects correlation with discriminant function 1.

^b^F2 reflects correlation with discriminant function 2.

^c^Scores for app usage items range from −2 (totally disagree) to +2 (totally agree).

There was a multivariate main effect of app use status (*F*_14,3348_=86.28, Wilk λ=0.54, *P*<.001, partial η^2^=0.27). The univariate tests revealed significant differences between means for all app experience items, which progressed linearly through the three profiles (Table 2). In comparison with previous users and informed nonusers, current app users believed that apps were easy to understand and retrieve information from; helped achieve their goals; were less boring to use; and required less time to understand. The same pattern of differences was found when comparing previous users with informed nonusers. In addition, current users and previous users had higher BMI and were younger as compared with informed nonusers.

## Discussion

### Distributions of Profiles

The results showed that little over only 5% of participants could be classified as previous users, while a much larger proportion could be classified as informed nonusers and the largest part of the study sample could be classified as current walking app users. These distributions are in line with earlier distributions regarding fitness app usage in German adults [19], which demonstrated that current users and informed nonusers were the most prevalent profiles. Interestingly, these distributions do not completely reflect the distributions found for nutrition apps in that same sample. Only a small proportion was classified as current nutrition app users, while a somewhat larger proportion was classified as previous nutrition app users [19]. The reasons for these discrepant proportions across nutrition and fitness or walking apps are unclear, but they may be explained by the fact that walking or fitness apps more unobtrusively collect, monitor, and report information regarding either walking (eg, distance and number of steps) or fitness performance (heart rate and heart rate variability), without the need for any input from the user to collect and present this information. In contrast, nutrition apps can mostly only collect and monitor nutrition intake via input from the user, such as textual input of items consumed. Such active involvement in the collection and monitoring of nutrition-related information may put off users from keeping engaged with the nutrition app.

It should also be noted that this study showed higher proportions of current app usage than those reported in other studies, where generally around one-fifth to one-quarter of the study population used apps [1,2]. Although this may be due to the fact that participants who did not use walking apps or did not know about walking apps were excluded, it may reflect increased interest in walking specifically or physical activity in general among those participating in walking events. Indeed, enhanced interest in a subject is known to stimulate learning and information search on that subject [41], both of which can be achieved through health apps [27]. It should also be noted that another recent study in the Dutch population on physical activity app usage found a relatively high proportion of app use [42], which could also indicate that the Dutch population may, in general, be more likely to use physical activity apps.

### Demographic and Behavioral Variables as Predictors of Profiles

The results demonstrated that current and previous walking app users not only were much younger than informed nonusers, but also had much higher BMI than informed nonusers. Furthermore, current users were more likely to have an unhealthy weight status (either being overweight or obese) than informed nonusers. These latter findings blend in with previous research in Dutch adults demonstrating that physical activity app users are more likely to report weight loss than nonusers [13]. They also blend in with previous intervention research demonstrating positive effects of app-based technologies on weight and waist circumference [43,44] and correlational research showing that app users are more likely to report more chronic conditions [2] and weight loss [1]. The exact role of walking app usage in weight loss is unclear, but it may suggest that people with an unhealthy BMI may use walking apps more often to obtain the benefits of walking apps. Future research is needed to understand which exact functions in walking apps are used by people with an unhealthy BMI to support them in their weight loss attempts. For instance, walking apps could be used to monitor physical activity levels, set personal and adaptable goals, or monitor progress toward one’s walking or weight goal, or could provide an opportunity to receive social support on weight loss through their use.

It should also be noted that this study did not find differences in gender and educational status between the three profiles. This contrasts previous work on mobile health app usage, where occasionally males and more often individuals with lower education [1] or individuals with lower healthy literacy [2] have been found to be less likely to use health apps. However, these earlier studies have collapsed various health-related activities, including smoking control, blood pressure control, medication intake, physical activity, and diet, into their assessments of health app usage [1,2], which prohibits a more fine-grained view of the relation between health behaviors, app usage, and demographics. For instance, there are clear gender differences in adherence to specific health behaviors (eg, walking and vegetable consumption) [45,46], yet these behaviors are often collapsed in studies on health app usage. For instance, Carrol et al [1] investigated app usage for “health-related reasons” and linked differences in app usage to differences in the intention to increase (1) fruit intake, (2) vegetable intake, and (3) physical activity and the intention to decrease (1) soda consumption and (2) weight. Although their findings demonstrated that males were more likely to use these health apps, it is unclear for which specific behaviors these apps were used by those males. Our findings indicate that focus on more specific behaviors in health app usage studies is prudent to better understand the interplay between health behaviors, app usage, and demographics.

When looking at the behavioral predictors, the findings for physical activity levels were mostly in line with previous work, that is, current walking app users were 40% more likely to walk at least 5 days per week and at least 30 minutes per session than informed nonusers. Previous work on understanding usage patterns of both general health apps [1,2] and more behavior-specific apps [47] commonly demonstrated healthier lifestyle patterns for app users. When comparing current users and previous users only, there were some interesting findings regarding previous use duration and frequency. Specifically, among current users, two-thirds reported having used a walking app for more than a year. In contrast, among previous users, this proportion was little over one-third. Perhaps more interesting was the substantial difference in app use frequency between current and previous users. Almost 60% of current users reported always using a walking app during a walking session, whereas less than a quarter of previous users reported always using a walking app. This could potentially indicate a relevant opportunity to enhance walking app user status by stimulating individuals to use their walking app during each and every walking session. Habit formation work could be an interesting area, and future research could consider the inclusion of survey-based habit measures to understand how and when one’s walking app usage becomes habitual [48].

### App Experience Variables as Predictors of Profiles

The strongest app use experience predictors of profiles were boredom and goal attainment. Participants who were classified as current users perceived the apps to be more helpful in achieving their walking goal and to be less boring to use compared with nonusers, with previous users also perceiving the apps to be more helpful for goal attainment and less boring compared with informed nonusers. These differences were of a large effect size and are particularly informative for walking app design features. Walking app developers should pay particularly close attention to avoid boring elements in their apps and to include aspects that foster walking goal attainment [49]. For instance, boredom has been defined as a state of “lack of stimulation” [50] and as the “aversive experience of wanting but being unable to engage in satisfying activity” [51], which may be alleviated by creating stimulating, engaging, and enjoyable tasks and environments [52]. Likewise, goals are more likely to be achieved when the set goals are both specific and relatively difficult to achieve [22,53]. Thus, walking app developers should consider creating engaging app environments that foster goal attainment. The results also showed significant differences across the three profiles for how easy it was to understand how to use an app, how long it took for people to understand how an app works, and the ease with which information could be derived from an app, with the highest mean scores for current users and the lowest scores for informed nonusers. There is some evidence that this lack of ease could be related to an overload of cognitive systems by the way the information is presented in competing modalities [54]. This overload can potentially be subverted by presenting information in such a way that textual and visual formats enhance, rather than interfere with, information processing [55]. Thus, a clear understanding of how people process information in mobile apps would be needed to enhance ease of use of health apps.

### Limitations

A few limitations need to be mentioned. First, the correlational and cross-sectional design of this study prohibits drawing conclusions about causality between walking app usage, walking, and weight status. A more thorough understanding of the role of walking apps (and health apps in general), walking, and weight status would preferably require longitudinal studies whereby the dynamics of walking, app usage, and weight status are investigated in, for instance, cross-lagged panel designs. Second, the participants were recruited from a public walking event. It may be that the included participants were not an accurate reflection of the general Dutch population in terms of motivation and interest in physical activity and related apps, even though the proportion of participants meeting physical activity recommendations in this study was lower than in other studies on mobile apps and physical activity [1], including a population-based sample [2]. Indeed, when comparing the proportion of app users in this study and in the aforementioned studies [1,2], our study had a much higher proportion of app users. Therefore, caution is needed to generalize these findings. Third, the logistic regression models had very low explanatory value, which is likely related to the absence of additional predictor variables, such as health literacy [2]. Fourth, not all instruments used in this study were validated, and they were mostly derived from more general frequency/duration instruments. Finally, there was an overrepresentation of females in the study sample, which makes generalization to the population at large problematic. It may have been the reason why no gender effects were found on walking app usage.

### Conclusions

Despite these limitations, this study demonstrates a few important insights that should help health practitioners to promote walking behavior, as well as help app developers to focus on important media-related technologies that foster walking app usage. It also demonstrates the need to study the predictors of behavior-specific apps, such as walking apps, rather than broader behavioral categories to better understand not only the demographic profiles of people who use those behavior-specific apps, but also how people evaluate those specific apps.

## Abbreviations

DFA: discriminant function analysis
OR: odds ratio
